# Retrospective analysis of clinical features and prognosis of nasopharyngeal carcinoma in children and adolescents

**DOI:** 10.3389/fped.2022.939435

**Published:** 2022-09-16

**Authors:** Tianyu Gong, Yupeng Liu, Huiqun Jie, Min Liang, Wenjin Wu, Jingrong Lu

**Affiliations:** ^1^Department of Otorhinolaryngology Head and Neck Surgery, Xinhua Hospital, Shanghai Jiao Tong University School of Medicine, Shanghai, China; ^2^Ear Institute, Shanghai Jiao Tong University School of Medicine, Shanghai, China; ^3^Shanghai Key Laboratory of Translational Medicine on Ear and Nose Diseases, Shanghai, China

**Keywords:** nasopharyngeal carcinoma, child, adolescent, drug therapy, radiotherapy, prognosis

## Abstract

**Objectives:**

To investigate the clinical characteristics and prognosis of nasopharyngeal carcinoma (NPC) in children and adolescents in different age groups.

**Materials and methods:**

The clinical data of 51 patients with NPC aged ≤ 18 years who were treated in Xinhua Hospital Affiliated to Shanghai Jiao Tong University School of Medicine from January 2012 to May 2017 were retrospectively analyzed. The patients were divided into children group (≤12 years old) and adolescent group (12–18 years old) with 12 years old as the boundary. The clinical characteristics, diagnosis, treatment, and prognosis of the children and adolescent groups were compared.

**Results:**

The symptoms of the first diagnosis in the children group were mainly nasal congestion (*P* = 0.043) and ear symptoms (*P* = 0.008). The diagnosis rate of nasopharyngeal biopsy in the children group was lower (*P* = 0.001), while the rate of diagnosis of cervical mass biopsy was significantly higher than that in the adolescent group (*P* = 0.009). The proportion of keratinizing squamous cell carcinoma of the children group was higher than that of the adolescent group (*P* = 0.006). There was no significant difference in TNM stage and risk stratification between the two groups, but the number of cases in the III-IVa children group who received induction chemotherapy + concurrent chemoradiotherapy was less than that in the adolescent group (*P* = 0.013). The proportion of radiotherapy in the upper and lower cervical lymph node drainage areas was lower than that in the adolescent group (*P* = 0.001). The percentage of recurrence and metastasis in the children group was higher than that in the adolescent group (*P* = 0.026).

**Conclusion:**

The diagnosis in the children group depended on endoscopic biopsy and neck mass biopsy, and the proportion of keratinizing squamous cell carcinoma was higher. The number of cases of induction chemotherapy and concurrent chemoradiotherapy in the children group was less than that in the adolescent group, and the proportion of radiotherapy in the upper and lower cervical lymph node drainage areas was lower than that in the adolescent group. Clinically, it is necessary to improve the understanding of the clinical characteristics of children with NPC and take appropriate treatment strategies.

## Introduction

Nasopharyngeal carcinoma (NPC) is one of the most common malignant tumors of the head and neck in adolescents (1, 2). Nasopharyngeal carcinoma accounts for about one-third of upper airway malignancies in children and adolescents, and is rare in children under the age of 10. The incidence rate is approximately 0.8 per 1 million people aged 10–14 years and 1.3 per 1 million people aged 15–19 years (3, 4). More than 90% of NPC cases in children and adolescents have invasive symptoms at the onset, and more than 80% have clinical stage III/IV (5, 6), which is higher than the clinical stage of NPC in adults, but the proportion of distant metastasis is lower than that in adults (7). With the application of comprehensive treatment strategies such as radiotherapy and chemotherapy, the prognosis of children and adolescents with NPC has improved significantly. The 5-year survival rate is greater than 80%, and the overall treatment effect is better than that of adult NPC (8).

Most of the existing studies have focused on adolescents, however, the prevalence of NPC in children has not been well described (7, 9). The clinical features, diagnosis, treatment, and prognosis of children with NPC younger than 12 years of age are not yet clear. It is necessary to compare the differences of clinical characteristics of NPC in the children and adolescents. Therefore, in this study, we retrospectively analyzed 51 children and adolescents with NPC in our hospital by age group, and explored the differences in clinical manifestations, TNM stages, recurrence risk levels, and different characteristics of children and adolescents with NPC.

## Patients and methods

### Ethical approval

The study was approved by the medical ethical committee of Shanghai Jiao Tong University School of Medicine (No. XHEC-D-2022-166).

### Setting

The children were divided into the children group (≤12 years old) and the adolescent group (12–18 years old) with 12 years old as the boundary.

### Subjects

The clinical data of 51 children with NPC aged ≤ 18 years who were treated in Xinhua Hospital Affiliated to Shanghai Jiao Tong University School of Medicine from January 2012 to May 2017 were collected. The differences in the clinical characteristics, diagnosis, treatment, and prognosis of the children and adolescent groups were compared.

### Inclusion and exclusion

Inclusion criteria were as follows: (1) Patients aged ≤ 18 years; (2) Patients who initially diagnosed with nasopharyngeal carcinoma and received complete treatment at Xinhua Hospital; (3) Patients who voluntarily participated in this study and signed the informed consent form; (4) Patients who underwent regular follow-up and had complete follow-up information.

### Intervention

The diagnosis of NPC patients was based on symptoms, and the results of nasopharyngeal biopsy, cervical lymph node biopsy and histopathological examination. Confirmed cases were evaluated according to the 2017 International Standards for Diagnosis and Treatment (10). Patients in stage II were treated with concurrent nasopharyngeal chemoradiotherapy, and those in stage III-IVa in the children group were treated with the combination of induction chemotherapy and concurrent chemoradiotherapy.

All patients received intensity-modulated radiation therapy (IMRT) as the primary treatment. IMRT planning and implementation techniques were performed as described previously (2, 5). The gross tumor volume (GTV) was defined as the primary tumor and enlarged lymph nodes. Radiotherapy was carried out for nasopharyngeal lesions and metastatic lymph nodes in the neck; besides, the upper cervical lymph node drainage area and the lower cervical lymph node drainage area in some cases were treated with radiotherapy as well. The prescribed doses of radiotherapy were divided into 66–70, 64–70, 60–62, and 54–56 Gy. All patients were treated 30–33 times in total, with the frequency of once a day for 5 days a week.

According to their conditions of the patients in two groups, different chemotherapy regimens were administered, including TP (docetaxel and cisplatin), FP (fluorouracil plus cisplatin), GP (gemcitabine and cisplatin), and docetaxel. During the radiotherapy, all the patients were assessed weekly. However, after the radiotherapy, patients were followed up every 3 months for the 1st and 2nd year, every 6 months from the 3rd to the 5th year, and annually thereafter. Besides, the adverse effects and complications after radiotherapy and chemotherapy, as well as the prognosis at 2 years and at 5 years were recorded in the following up.

### Data collection

Data were collected from all the patients, including the gender, initial symptoms and confirmatory examinations, TNM stage, risk level, histological type (Table 1); as well as regimens for the treatment, complications, and the prognosis (Table 2). The TNM staging and risk levels were assessed according to the 2017 International Standards of Cancer Staging (Table 1) (10).

**TABLE 1:** Clinical data of nasopharyngeal carcinoma in children and adolescents.

	Children	Adolescents	χ^2^ (*t*)	*P*	Total
	*n* = 19	*n* = 32			*n* = 51
Sex			0.008	0.928^a^	
Male	17 (89.5%)	27 (84.4%)			44 (86.3%)
Female	2 (10.5%)	5 (15.6%)			7 (13.7%)
**Local symptoms**					
Nasal congestion	7 (36.8%)	3 (9.4%)	4.096	0.043^a^	10 (19.6%)
Nosebleeds	2 (10.5%)	12 (37.5%)	3.106	0.078	14 (27.5%)
Ear symptoms	3 (15.8%)	17 (53.1%)	6.972	0.008	20 (39.2 %)
Neck lumps	14 (73.7%)	19 (59.4%)	1.069	0.301	33 (64.7%)
Sleep snoring	5 (26.3%)	3 (9.4%)	1.465	0.226^a^	8 (15.7%)
**Invasion symptoms**					
Local nerve invasion: headache, facial numbness	8 (42.1%)	15 (46.9%)	0.110	0.741	23 (45.1%)
Eye symptoms: diplopia, fixation, blindness	2 (10.5%)	3 (9.4%)	0.000	1.000^a^	5 (9.8%)
**Metastatic symptoms**					
Lung/bone/distant lymph node metastasis	2 (10.5%)	0 (0.0%)	3.506	0.134^b^	2 (3.9%)
**Diagnosed**					
Nasopharyngeal biopsy	12 (63.2%)	32 (100.0%)	10.731	0.001^a^	44 (86.3%)
Neck lymph node aspiration/biopsy	11 (57.9%)	7 (21.9%)	6.773	0.009	18 (35.3%)
Both tests	4 (21.1%)	7 (21.9%)	0.000	1.000^a^	11 (21.6%)
Pathology			7.569	0.006^a^	
KSCC	9 (47.4%)	3 (9.4%)			12 (23.5%)
Non-KSCC	10 (52.6%)	29 (90.6%)			39 (76.5%)
**EBV**					
Positive case	19 (100.0%)	32 (100.0%)	/	/	51 (100.0%)
T stage			/	0.695^c^	
T1	1 (5.3%)	1 (3.1%)			2 (3.9%)
T2	5 (26.3%)	2 (6.3%)			7 (13.7%)
T3	3 (15.8%)	14 (43.8%)			17 (33.3%)
T4	10 (52.6%)	15 (46.9%)			25 (49.0%)
N stage			/	0.195^c^	
N1	2 (10.5%)	2 (6.3%)			4 (7.8%)
N2	9 (47.4%)	24 (75.0%)			33 (64.7%)
N3	8 (42.1%)	6 (18.8%)			14 (27.5%)
M stage			/	0.064^c^	
M0	17 (89.5%)	32 (100.0%)			49 (96.1%)
M1	2 (10.5%)	0 (0.0%)			2 (3.9%)
Risk level			/	0.142^c^	
I	0 (0.0%)	0 (0.0%)			0 (0.0%)
II	1 (5.3%)	1 (3.1%)			2 (3.9%)
III	5 (26.3%)	15 (46.9%)			20 (39.2%)
IVa	11 (57.9%)	16 (51.6%)			27 (52.9%)
IVb	2 (10.5%)	0 (0.0%)			2 (3.9%)

^a^Correction for continuity.

^b^Fisher exact test.

^c^Mann-Whitney U-test. KSCC, keratinizing squamous cell carcinoma; Non-KSCC, non-keratinizing squamous cell carcinoma. TNM staging and risk levels were assessed according to The Eighth Edition (2017) AJCC Cancer Staging Manual.

**TABLE 2:** Treatment and follow-up of nasopharyngeal carcinoma in children and adolescents.

	Children	Adolescents	χ^2^ (*t*)	*P*	Total
	*n* = 19	*n* = 32			*n* = 51
Treatment regimen			7.468	0.013^b^	
I: Nasopharyngeal radical radiotherapy + cervical preventive radiotherapy	0 (0.0%)	0 (0.0%)			0 (0.0%)
II: Nasopharyngeal concurrent chemoradiotherapy	5 (26.3%)	2 (6.3%)			7 (13.7%)
III-IVa: Induction chemotherapy + concurrent chemoradiotherapy	12 (63.2%)	30 (93.8%)			42 (82.4%)
IVb: Supportive care + systemic palliative chemotherapy and palliative radiotherapy	2 (10.5%)	0 (0.0%)			2 (3.9%)
**Radiotherapy**					
Nasopharyngeal lesions + nasopharyngeal CTV	16 (84.2%)	22 (68.8%)	0.797	0.372^a^	38 (74.5%)
Metastasized lymph nodes in the neck	13 (68.4%)	23 (71.9%)	0.069	0.794	36 (70.6%)
Upper neck lymph node area	5 (26.3%)	25 (78.1%)	13.211	0.000	30 (58.8%)
Lower neck lymph node area	9 (47.4%)	30 (93.8%)	11.792	0.001^a^	39 (76.5%)
Other metastases	2 (10.5%)	0 (0.0%)	3.506	0.134^b^	2 (3.9%)
**Chemotherapy drugs**					
TP	12 (63.2%)	20 (62.5%)	0.002	0.963	32 (62.7%)
FP	13 (68.4%)	17 (53.1%)	1.152	0.283	30 (58.8%)
GP	2 (10.5%)	1 (3.1%)	0.221	0.638^a^	3 (5.9%)
Docetaxel + platinum	2 (10.5%)	2 (6.3%)	0.000	0.992^a^	4 (7.8%)
Targeted therapy	1 (5.3%)	2 (6.3%)	0.000	1.000^a^	3 (5.9%)
**Complication**					
Dry mouth	12 (63.1%)	27 (84.3%)	1.920	0.166^a^	40 (78.4%)
Skin tissue fibrosis	6 (31.6%)	21 (65.6%)	5.547	0.019	27 (52.9%)
Difficulty opening mouth	3 (15.7%)	15 (46.9%)	5.044	0.025	18 (35.2%)
Hearing loss	8 (42.2%)	14 (43.8%)	0.013	0.909	22(43.1%)
Myelosuppression	6 (31.6%)	18 (56.3%)	2.913	0.088	24 (47.1%)
Prognosis			/	0.026^c^	
Cured	7 (36.8%)	20 (62.5%)			27 (52.9%)
Under control	7 (36.8%)	11 (34.4%)			18 (35.3%)
Recurrence and metastasis	4 (21.1%)	1 (3.1%)			5 (9.8%)
Dead	1 (5.3%)	0 (0.0%)			1 (2.0%)

^a^Correction for continuity.

^b^Fisher exact test.

^c^Mann-Whitney U-test. CTV, clinical target volume; TP, Paclitaxel + Cisplatin; FP, Fluorouracil + Cisplatin; GP, Gemcitabine + Platinum.

### Data analysis

Statistical analysis of the data was performed with SPSS software (SPSS, 26.0). Enumeration data were expressed as rate (%), and the chi-square test or Fisher’s exact test was used to compare categorical variables between children and adolescents, such as gender, newly diagnosed symptoms, confirmatory examination, pathological results, complications, metastasis, radiotherapy, chemotherapy and other treatments. The Mann-Whitney *U*-test was used to assess rank variables between the two groups, including tumor stage, risk level, and prognosis. Metastasis- and recurrence-free survival (RFS) was assessed by the Kaplan-Meier method. The significance level was set at *P*-value<0.05.

## Results

Children are divided into preschool age and school age according to their age characteristics. Among the 51 cases in this group, 19 cases were in the preschool age group of ≤ 12 years old, and 32 cases were in the school age group from 12 years old to 18 years old. The proportion of male patients in the two groups of children and adolescents (17 cases, 89.5%; 27 cases, 84.4%) was significantly higher than that of female patients, and there was no statistical difference between the two groups (χ^2^ = 0.008, *P* = 0.928) (Table 1). There was no significant difference in the first diagnosis symptoms of nosebleed, neck mass and sleep snoring between the two groups (χ^2^ = 3.106, *P* = 0.078; χ^2^ = 1.069, *P* = 0.301; χ^2^ = 1.465, *P* = 0.226). However, there were significant statistical differences in nasal congestion and ear symptoms between the two groups. The number of nasal congestion symptoms in the children group was higher than that in the adolescent group (7 cases, 36.8%; 3 cases, 9.4%) (χ^2^ = 4.096, *P* = 0.043). The number of ear symptoms in the adolescent group was significantly higher than that in the children group (17 cases, 53.1%; 3 cases, 15.8%) (χ^2^ = 6.972, *P* = 0.008), 13 patients in the adolescent group had tinnitus and/or hearing loss and 4 patients had ear fullness, while in the children group 2 patients had tinnitus and/or hearing loss and 1 patient had secretory otitis media (Table 1). There were no significant differences in local invasion (χ^2^ = 0.110, *P* = 0.741), ocular symptoms (χ^2^ = 0.000, *P* = 1.000) and metastasis (χ^2^ = 3.506, *P* = 0.134) between the two groups (Table 1).

In the children group, the proportion of nasopharyngeal biopsy as a diagnostic test was lower than that in the adolescent group (12 cases, 63.2%; 32 cases, 100%) (χ^2^ = 10.731, *P* = 0.001), while the proportion of children diagnosed by neck mass biopsy was higher than that of adolescents (11 cases, 57.9%; 7 cases, 21.9%) (χ^2^ = 6.773, *P* = 0.009) (Table 1). In the pathological diagnosis, the proportion of keratinizing squamous cell carcinoma in the children group was higher than that in the adolescent group (9 cases, 47.4%; 3 cases, 9.4%) (χ^2^ = 7.569, *P* = 0.006) (Table 1). The positive rate of EBV at diagnosis was 100% in both groups.

In terms of TNM staging in children and adolescents, the proportion of T3 in children was slightly lower than that in adolescents (3 cases, 15.8%; 14 cases, 43.8%), and the proportion of N2 in the children group was slightly lower than that in the adolescent group (9 cases, 47.4%; 24 cases, 75%). In contrast, the proportion of M1 in the children group was slightly higher than that in the adolescent group (2 cases, 10.5%; 0 cases, 0.0%). However, there was no significant difference in TNM staging between the two groups (*P* = 0.695; *P* = 0.195; *P* = 0.064). In terms of percentage values, the proportion of III in the children group was slightly lower than that in the adolescent group (5 cases, 26.3%; 15 cases, 46.9%), and there was no significant difference in the risk level between the two groups (*P* = 0.142) (Table 1).

In the children group and the adolescent group, concurrent nasopharyngeal chemoradiotherapy was used for stage II cases (5 cases, 26.3%; 2 cases, 6.3%). Among stage III-IVa cases, the number of children receiving induction chemotherapy and concurrent chemoradiotherapy was less than that of the adolescent group (12 cases, 63.2%; 30 cases, 93.8%), and there was a significant difference between the two groups (χ2 = 7.468, *P* = 0.013) (Table 2). There was no significant difference in the proportion of nasopharyngeal lesions and nasopharyngeal CTV (16 cases, 84.2%; 22 cases, 68.8%) and neck metastases (13 cases, 68.4%; 23 cases, 71.9%) receiving radiotherapy in the two groups (χ^2^ = 0.797, *P* = 0.372; χ^2^ = 0.069, *P* = 0.034). The proportion of radiotherapy in the upper cervical lymph node drainage area (5 cases, 26.3%; 25 cases, 78.1%) and lower cervical lymph node drainage area (9 cases, 47.4%; 30 cases, 93.8%) in the children group was lower than that in the adolescent group, and there was a statistically significant difference (χ^2^ = 13.211, *P* = 0.000; χ^2^ = 11.792, *P* = 0.001) (Table 2). There was no significant difference in the proportion of chemotherapy regimens such as TP, FP, GP, and docetaxel between the two groups (Table 2).

There was no significant difference in the proportion of dry mouth, hearing loss, and bone marrow suppression in the children group after radiotherapy and chemotherapy (χ^2^ = 1.920, *P* = 0.166; χ^2^ = 0.013, *P* = 0.909; χ^2^ = 2.913, *P* = 0.088), while the proportion of skin fibrosis (6 cases, 31.6%; 21 cases, 65.6%) and mouth opening difficulty (3 cases, 15.7%; 15 cases, 46.9%) in the children group was lower than that in the adolescent group (χ^2^ = 5.547, *P* = 0.019; χ^2^ = 5.044, *P* = 0.025) (Table 2).

During the follow-up of children and adolescents, the number of cured cases in the children group was less than that in the adolescent group (7 cases, 36.8%; 20 cases, 62.5%), and the proportion of local control in the two groups was similar (7 cases, 36.8%; 11 cases, 34.4%). The percentage of recurrence and metastasis in the children group was higher than that in the adolescent group (4 cases, 21.1%; 1 case, 3.1%), and the prognosis of the two groups was significantly different (*P* = 0.026) (Table 2).

**FIGURE 1:**
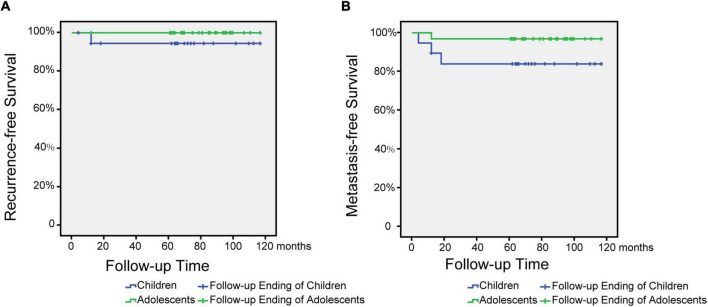
Metastasis and recurrence-free survival time after treatment for children and adolescents with nasopharyngeal carcinoma. **(A)** Relapse-free survival time after treatment for children and adolescents with NPC. **(B)** Metastasis-free survival time after treatment for children and adolescents with NPC.

When the children group was followed up for 2 years, 7 cases were cured (36.8%), 7 cases were locally controlled (36.8%), 1 case was relapsed (5.3%), 3 cases were metastasized (15.7%), and 0 cases died (0.0 %), and when the children group was followed up for 5 years, 7 cases (36.8%) were cured, 7 cases (36.8%) were locally controlled, 1 case was relapsed (5.3%), 2 cases were metastasized (10.5%), and 1 case died (5.3%). During the 2-year follow-up of the cases in the adolescent group: 20 cases (62.5%) were cured, 11 cases (34.4%) were locally controlled, 0 cases (0.0%) recurred, 1 case (5.3%) metastasized, and 0 cases died (0.0 %), the data remained unchanged at 5 years of follow-up. Kaplan-Meier survival curves were used to compare children and adolescents with NPC in different age groups (≤12 years old and 12–18 years old) under univariate influence. The blue curve was the children group and the green curve was the adolescent group (Figure 1).

## Discussion

This study mainly included children and adolescents who were less than or equal to 18 years of age with NPC. The patients were divided into children (≤12 years old) and adolescents (12–18 years old) according to their growth and development stages. The clinical characteristics, treatment options and prognosis of the two groups were compared and analyzed.

Approximately 60–90% of patients with NPC reported a neck mass as the first symptom at the initial diagnosis. In the subsequent course of the disease, symptoms of the noses (30–70%) and ears (20–45%) have been found to be more common (8, 11–14). In our study, neck masses were frequently presented in both groups (73.7% in the children group vs. 59.4% in the adolescent group), and the symptom of epistaxis in the adolescent group was more usual compared to the children group (37.5% vs. 10.5%). However, the distribution of neck masses and epistaxis between groups was not significantly different, suggesting that they were very common in the initially diagnosed patients with NPC (regardless of the children or the adolescents) (Table 1). Additionally, we also found several symptoms of initially diagnosed patients with NPC significantly different between groups. Compared to the adolescent group, the symptoms of nasal congestion (36.8% vs. 9.4%) were more common in the children group; however, the symptoms of ears (15.8% vs. 53.1%) were less common in the children group. These findings indicated that for the children, nasopharyngeal examination should be considered at the first diagnosis of NPC; while for the adolescent group, the possibility of auditory lesions should be evaluated at the first diagnosis of NPC.

As previously reported, the children and adolescents with NPC often have localized invasive symptoms, including the headache (11–32%), cranial nerve deficits (5–22%), and ocular symptoms (26%) (13, 15). Local invasion of the nerves and eyes are often recognized at the first diagnosis. The proportion of cranial nerve invasion in our study was greater than 40%, and that of ocular symptoms were about 10% in both groups, which was consistent with the number reported in other literatures. Notably, at the first diagnosis, two cases with distant metastases were found in the children group but no distant metastasis was found in the adolescent group, which may be attributed to the easier spreading and metastasis of the lesions due to the lack of chief complaints of the children (Table 1).

The diagnostic methods of nasopharyngeal carcinoma mainly include the nasal endoscopy, biopsy, examination of plasma EBV DNA, imaging, Chen et al. (16). Considering some tumors at the early stage are not obvious under nasal endoscopy, magnetic resonance imaging (MRI) and positron emission tomography/computed tomography (PET/CT), neck puncture are often used for the diagnosis of NPC (17). The children were most often diagnosed based on the results the biopsy of neck masses (57.9%), while the adolescents were more frequently diagnosed according to the results of nasopharyngeal biopsy (100%) in our study. Thus, to validate the diagnosis, we should pay more attention to the biopsy of neck masses in the children and nasopharyngeal biopsy in the adolescents, the latter of which is similar to the diagnostic method of nasopharyngeal carcinoma in adults (18).

The keratinizing squamous cell carcinoma (KSCC) has been found to account for less than 5% of NPCs, while Non-KSCC and anaplastic Carcinoma account for more than 90% of NPCs (19, 20). In our study, the proportion of KSCC in the children group was significantly higher than that in the adolescent group (47.4% vs. 9.4%) (*P* = 0.006) (Table 1), suggesting that the pathological types of NPC in the children were different from those in the adolescents (21, 22). The proportion of KSCC in children and adolescents in our study was significantly higher than that in other studies, suggesting that the patients in our study may come from an area with a high incidence of KSCC. The poor radiosensitivity of KSCC suggested a poor prognosis in the children with NPC. The positive rate of EBV was 100% in both groups at the time of diagnosis, suggesting that the serological detection of EBV might play an important role in the diagnosis of NPC in the children and adolescents.

The results of previous studies demonstrated that the proportion of stage III NPC was approximately 25–43.2%, while the proportion of stage IV NPC reached up to 53–72% (8, 15, 20, 23). In this study, approximately 25% of the children and adolescents had already been at Stage III of NPC when diagnosed, and more than 50% had been at Stage IV of NPC, indicating the lack of early diagnosis of NPC for these patients. In this study, the proportion of patients with stage IV NPC in the adolescent group (51.6%) was similar to that in the previous studies; comparatively, the proportion of patients with stage IV NPC in the children group was much higher (68.4%) than that in the previous studies, contributing to the poor prognosis finally.

Since NPC has been found to be highly sensitive to the radiotherapy, it has become the main treatment for metastatic NPC. Notably, the radiotherapy has been routinely used as the basis of the treatments in almost all cases in related studies (20). To decrease the dose of radiotherapy, it is recommended for the children with stage II to IVA NPC to receive the induction chemotherapy first, followed by concurrent chemoradiotherapy. In the patients with III-IVA NPC in this study, the proportion of patients who received induction chemotherapy followed by concurrent chemoradiotherapy in the children group was lower than that in the adolescent group (63.2% vs. 93.8%). The underlying reason may be that the physicians tended to adopt conservative strategies for the children group, resulting in inadequate treatment. Therefore, we suggest that for the children with NPC, the standard induction chemotherapy and concurrent chemoradiotherapy should be carried out in accordance with the Guidelines for the Diagnosis and Treatment of NPC in Children and Adolescents (2021 Edition).

In the previous studies, the incidence of hearing loss in children and adolescents after the radiotherapy and chemotherapy was approximately 35%, and the incidence of skin fibrosis was 33% (24), which was consistent with the results of this study. Besides, the incidence of myelosuppression in this study was about 47.1%; however, it was as high as 85% in other literature (25), possibly due to the higher proportion of children in our study.

The 5-year survival rate and the 3-year event-free survival rate of the patients in our study was 98.0 and 90.2%, respectively, which were consistent with the results of the previous studies. As reported, the 5-year survival rate of pediatric patients with NPC after treatment was be 78–97%, and the 5-year event-free survival rate was 7–592% (23, 26). These results suggest that the overall prognosis of the children and adolescents with NPC may be relatively good. During the follow-up, the proportion of the cured cases in the children group was less than that in the adolescent group (36.8% vs. 62.5%), and the proportion of local control in the two groups was similar. In addition, the percentage of recurrence and metastasis in the children group was higher than that in the adolescent group (21.1% vs. 3.1%), and the prognosis of the two groups was significantly different, suggesting that the prognosis for patients with NPC in the children group may be worse than that in the adolescent group.

Therefore, attention should be paid to the clinical characteristics of NPC in the children and adolescents, including the neck mass, symptoms of epistaxis, and localized invasive symptoms (such as the headache, cranial nerve deficits, and ocular symptoms). Puncture biopsy of the neck masses and endoscopic nasal biopsy are necessary, especially for the children with the symptoms of nasal congestion and for the adolescents with the symptoms in ears. Compared to the adolescent group, the worse prognosis in the children group could be related with the higher proportion of KSCC, the higher proportion of stage IV NPC, and the lower proportion of III-IVA NPC who received the induction chemotherapy and concurrent chemoradiotherapy.

There were several limitations in this study. This was a retrospective study, thus potential bias, such as selection bias should be considered. Besides, the patents included in this study were from one clinical center, which limited the representativeness of our study. In addition, the overall diagnosis, treatment, and prognosis of NPC in the children are still insufficient, and large-scale prospective data are needed for further exploration.

In conclusion, this study revealed the clinical characteristics of NPC in the children and adolescents less than 18 years old, which may provide the guidance and direction for future prospective studies.

## Data availability statement

The raw data supporting the conclusions of this article will be made available by the authors, without undue reservation.

## Ethics statement

The studies involving human participants were reviewed and approved by the Ethics Committee of Xinhua Hospital Affiliated to Shanghai Jiao Tong University School of Medicine. Written informed consent to participate in this study was provided by the participants’ legal guardian/next of kin.

## Author contributions

All authors listed have made a substantial, direct, and intellectual contribution to the work, and approved it for publication.
